# Associations of COVID-19 lockdown with gestational length and preterm birth in China

**DOI:** 10.1186/s12884-021-04268-5

**Published:** 2021-11-27

**Authors:** Moran Dong, Rui Qian, Jiaqi Wang, Jingjie Fan, Yufeng Ye, He Zhou, Brian Win, Eve Reid, Suijin Zheng, Yanyun Lv, Yudong Pu, Hanwei Chen, Juan Jin, Qingmei Lin, Xiaoyang Luo, Guimin Chen, Yumeng Chen, Zhongrong He, Guanhao He, Shouzhen Cheng, Jianxiong Hu, Jianpeng Xiao, Wenjun Ma, Tao Liu, Xiaozhong Wen

**Affiliations:** 1 Guangdong Provincial Institute of Public Health, Guangdong Provincial Center for Disease Control and Prevention, Guangzhou, 511430 China; 2 School of Public Health, Southern Medical University, Guangzhou, 510515 China; 3 Statistical Information Center for Health and Family Planning Bureau of Foshan, Foshan, 528000 China; 4 School of Public Health, Guangdong Pharmaceutical University, Guangzhou, 510080 China; 5 Department of Prevention and Health Care, Shenzhen Maternity & Child Healthcare Hospital, Southern Medical University, Shenzhen, 518028 China; 6 Guangzhou Panyu Central Hospital, Guangzhou, 511400 China; 7 Division of Behavioral Medicine, Department of Pediatrics, Jacobs School of Medicine and Biomedical Sciences, State University of New York at Buffalo, Buffalo, NY 14214 USA; 8 The Affiliated Houjie Hospital, Guangdong Medical University, Dongguan, 523945 China; 9 Affiliated Jiangmen Hospital of Sun Yat-sen University, Jiangmen, 529030 China; 10 Songshan Lake Central Hospital of Dongguan City, Dongguan, 523808 China; 11 Foshan Women and Children Hospital Affiliated to Southern Medical University, Foshan, 528000 China; 12 Department of Ophthalmology, Guangdong Provincial People’s Hospital, Guangzhou, 510000 China; 13 School of Public Health, Sun Yat-sen University, Guangzhou, 510080 China; 14 Nursing Department, The First Affiliated Hospital, Sun Yat-sen University, Guangzhou, 510080 China; 15 Department of Public Health and Preventive Medicine, School of Medicine, Jinan University, No.601 West, Huangpu Road, Tianhe District, Guangzhou, 510632 China

**Keywords:** COVID-19, Lockdown, Gestational length, Preterm, China

## Abstract

**Background:**

The effects of COVID-19 lockdown measures on maternal and fetal health remain unclear. We examined the associations of COVID-19 lockdown with gestational length and preterm birth (PTB) in a Chinese population.

**Methods:**

We obtained medical records of 595,396 singleton live infants born between 2015 and 2020 in 5 cities in Guangdong Province, South China. The exposed group (*N* = 101,900) included women who experienced the COVID-19 Level I lockdown (1/23–2/24/2020) during pregnancy, while the unexposed group (*N* = 493,496) included women who were pregnant during the same calendar months in 2015–2019. Cumulative exposure was calculated based on days exposed to different levels of emergency responses with different weighting. Generalized linear regression models were applied to estimate the associations of lockdown exposure with gestational length and risk of PTB (< 37 weeks).

**Results:**

The exposed group had a shorter mean gestational length than the unexposed group (38.66 vs 38.74 weeks: adjusted β = − 0.06 week [95%CI, − 0.07, − 0.05 week]). The exposed group also had a higher risk of PTB (5.7% vs 5.3%; adjusted OR = 1.08 [95%CI, 1.05, 1.11]). These associations seemed to be stronger when exposure occurred before or during the 23rd gestational week (GW) than during or after the 24th GW. Similarly, higher cumulative lockdown exposure was associated with a shorter gestational length and a higher risk of PTB.

**Conclusions:**

The COVID-19 lockdown measures were associated with a slightly shorter gestational length and a moderately higher risk of PTB. Early and middle pregnancy periods may be a more susceptible exposure window.

**Supplementary Information:**

The online version contains supplementary material available at 10.1186/s12884-021-04268-5.

## Background

The ongoing COVID-19 pandemic has spread throughout the world and affected billions of people [1, 2]. Various measures have been implemented around the world to control the pandemic, including restricting large social movements and gatherings, closing international and interstate borders, controlling travel, and implementing partial or full lockdown of cities and regions [3–5]. These measures have effectively controlled the spread of COVID-19 and reduced the anthropogenic emissions of air pollution [6], which have resulted in substantial health benefits [7]. However, these measures have also caused huge economic loss, unemployment, shortage of medical resources, and psychological stress [8–11], which may lead to adverse health outcomes [12, 13].

Pregnant women and fetuses may be susceptible populations to the effects of lockdown and restriction measures. A few studies have reported that the COVID-19 lockdown measures may increase the risk of adverse birth outcomes such as stillbirth and cesarean delivery [12, 13]. Preterm birth (PTB) is one of the most important adverse birth outcomes and a major cause of death in children under 5 years of age [14]. Several studies have examined the associations of COVID-19 lockdown measures with the risk of PTB, but the results were inconsistent [12, 13, 15–18]. A study in London reported an increase in the incidence of PTB during the COVID-19 pandemic period over the pre-pandemic period.^12^ Another study conducted in Nepal also observed a greater risk of PTB during the COVID-19 lockdown than before lockdown [12]. In contrast, studies conducted in Denmark and the Netherlands observed a substantial reduction in the risk of PTB during the COVID-19 periods than before lockdown [16, 17]. The other two studies conducted in China and Botswana did not find any significant association between the COVID-19 lockdown and the risk of PTB [13, 18]. The inconsistent findings across these studies may be attributable to differences in study design, sample size, demographic characteristics of study subjects, and socioeconomic developments of societies [19, 20].

Although the studies have preliminarily estimated the associations between COVID-19 lockdown and PTB, several research issues or gaps need to be addressed. First, the susceptibility of pregnant women to environmental factors largely depends on the stage of pregnancy [21, 22]. Previous studies estimated the overall rate of PTB in pregnant women exposed to COVID-19 lockdown measures [12, 13, 15, 16, 18, 23–25], but did not consider their pregnancy stage when lockdown occurred. This may lead to an underestimation of PTB risk during the lockdown if pregnant women with a gestational age > 36 weeks were also included. Second, lockdown intensity usually varied over time [20]. However, none of the previous studies considered the change in intensity of lockdown exposures. Third, previous studies have suggested a seasonal variation in the incidence of PTB [26, 27]. The seasonal effects should be considered in selecting the control periods for the COVID-19 lockdown. However, some previous studies applied the annual or multiple years’ average incidence of PTB as the reference [13, 16, 18], which might lead to biased findings. Fourth, the follow-up time (2–4 months) in previous studies was not long enough to capture the birth outcomes of pregnant women who experienced the lockdown in their early pregnancy [12, 13, 15, 16, 18].

To fill these research gaps, we comprehensively elucidated the association of the COVID-19 lockdown on gestational length and PTB risk in South China by quantifying the timing and intensity of exposure, considering seasonal effects, and allowing sufficient follow-up time. This study could provide in-depth insights to inform management practices regarding pregnancy and childbirth during and after lockdown.

## Methods

### Study design, settings, and subjects

**Fig. 1:**
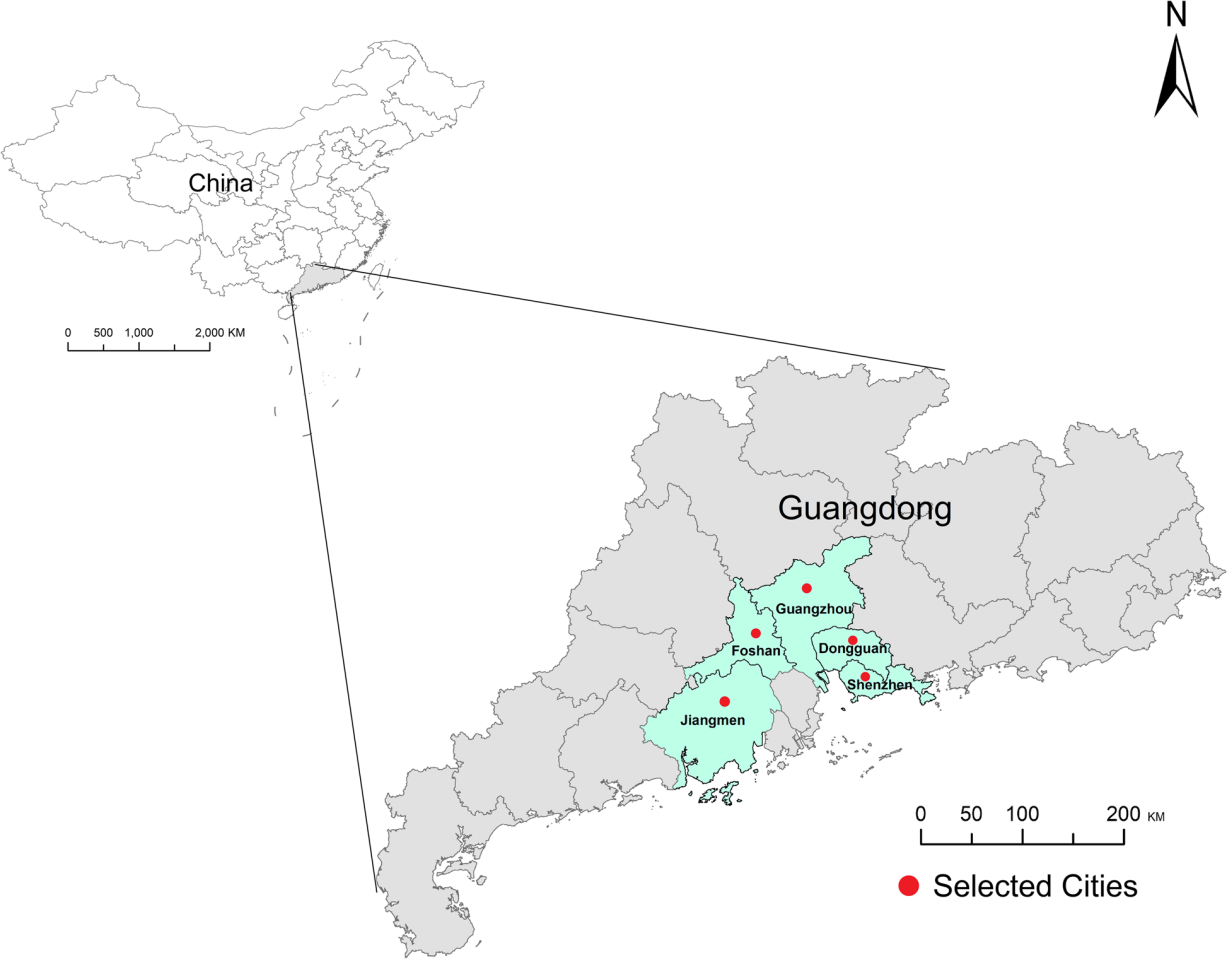
Geographic locations of the 5 study cities in Guangdong Province, South China

This study was a hospital-based retrospective study comparing the risk of PTB between the COVID-19 lockdown period in 2020 and the same periods in 2015–2019. We selected all hospitals in Foshan (*n* = 62) and several other hospitals in Guangzhou (*n* = 1), Shenzhen (*n* = 1), Dongguan (*n* = 2), and Jiangmen (*n* = 1) in Guangdong Province, South China, as study settings (Fig. 1 - Map). All hospital birth data from 1/1/2015 to 12/31/2020 were collected (*n* = 749,059). Birth records with multiple births (*n* = 27,659), stillbirths (*n* = 726), or missing information on key variables (*n* = 2883) were excluded. Moreover, 122,395 births were excluded because their pregnancy did not overlap with the COVID-19 lockdown in 2020 or the same calendar months in 2015–2019. Finally, 595,396 mother-newborn pairs were included. None of these women had a positive SARS-CoV-2 test result (Fig. S1 - Flowchart).

### Data collection

The following information on each birth was extracted from the hospital information system or birth record system: infant sex, date of birth, delivery type (vaginal or cesarean), gestational weeks (GW) at birth, maternal age, parity, pregnancy complications such as hypertensive disorders of pregnancy (HDP) and gestational diabetes mellitus (GDM), and major adverse pregnancy outcomes such as miscarriage and stillbirth. Geographic information system covariates (geographic map) come from the Data Center for Resources and Environmental Sciences (https://www.resdc.cn). We carefully checked the accuracy and quality of source data. Implausible values and outliers were either corrected or recoded as missing.

### Exposure assessment

The National Emergency Response Plan for Public Emergencies by the China State Council defined 4 levels of emergency response: Level I (extremely serious), Level II (serious), Level III (relatively serious), and Level IV (common) [28]. After the outbreak of COVID-19, the Guangdong Provincial Government announced a Level I response on 1/23/2020 and later degraded the response level to Level II and Level III on 2/24/2020 and 5/9/2020, respectively. The Level III response was maintained after 5/9/2020. During the Level I response, offices, shops, colleges, schools, childcare facilities, and all other non-essential institutions were shut down. Residents’ social activities and gatherings were rigorously restricted. Most of the workforce adapted to a new work-from-home mode due to traffic and mobility restrictions. Fewer restriction measures were implemented during the Level II and Level III responses. During the Level II response, crowded areas were temporarily closed and disinfected before reopening. During the Level III response, people’s lives gradually returned to normal. All shopping malls, supermarkets, hotels, restaurants, and other living areas were reopened with routine precautionary measures such as wearing masks and practicing social distancing (Table S1).

We defined the period with a Level I response (1/23-2/24/2020) as Level I lockdown. Women who were pregnant during the Level I lockdown period were defined as the exposed group (*N* = 101,900). Women who were pregnant during the same calendar months in 2015–2019 were defined as the unexposed group (*N* = 493,496). This served to control for the seasonal effect, as our data indicated a significant variation in PTB rate across calendar months of conception (Fig. S2).

To further explore the potential susceptible exposure window, we divided the exposed group into 11 subgroups according to their GW on 1/23/2020. We determined the day of conception based on the gestational length and date of birth. For example, pregnant women were recorded as the first group when the date of conception crossed with the Level I lockdown, and the second group were recorded as women with GWs less than 4 weeks on 1/23/2020. (Fig. S3). The gestational age of all women over 41 GWs was consistently recorded as the group of 41st GW. Similarly, the unexposed group was divided into correspondingly matching subgroups. With each pair of subgroups (exposed vs unexposed), we estimated the associations of lockdown exposure with gestational length and PTB.

**Fig. 2:**
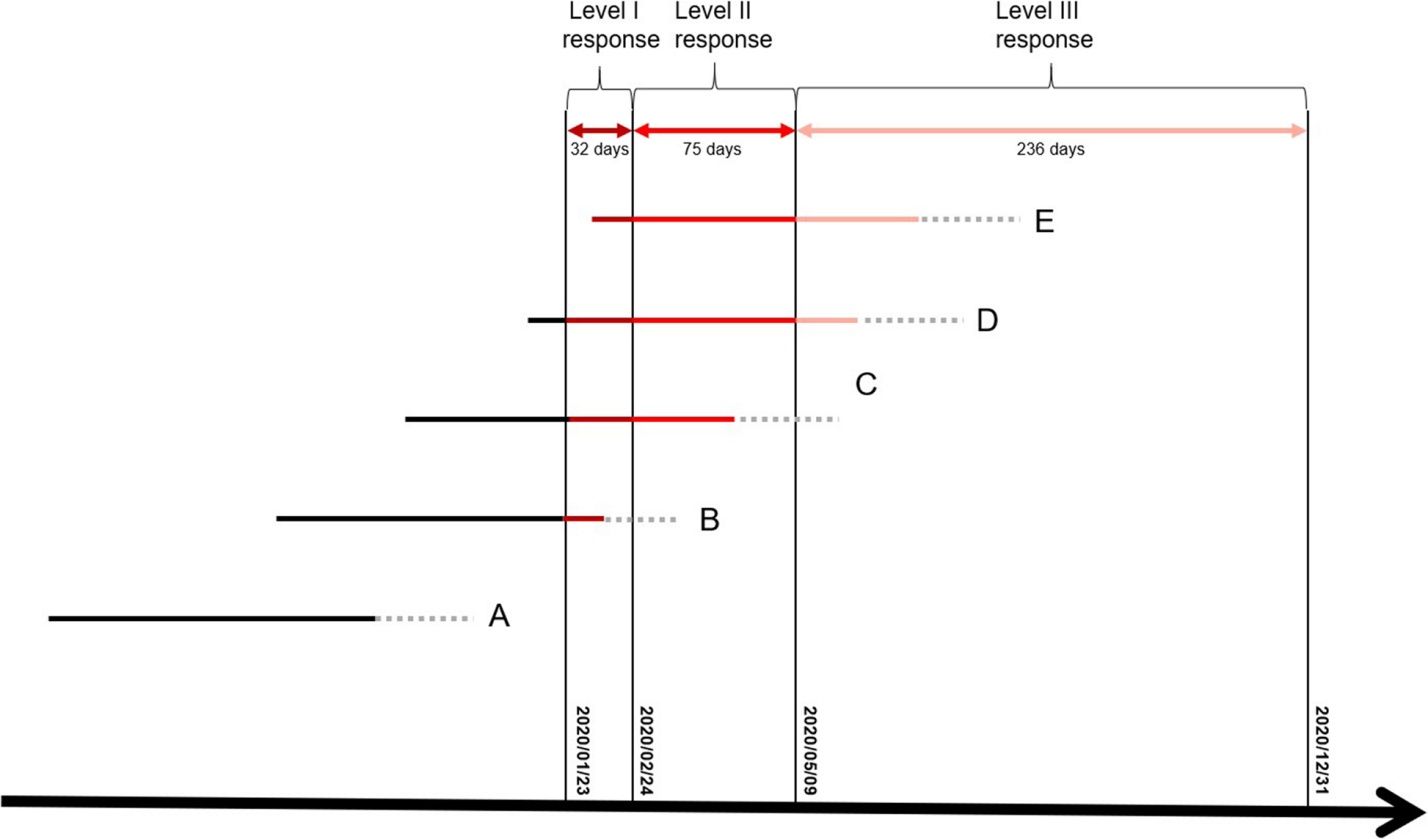
Approach to calculating individual cumulative exposure dose to lockdown in the first 22 GWs.  : Weeks after 22 GWs. Note: A, B, C, D and E represent subgroups of pregnant women with different GWs during the Level I lockdown; We assigned a weighting value of 3 to the days with Level I response, 2 to the days with Level II response, 1 to the days with Level III response, and 0 to days before lockdown (no exposure)

Restriction measures during the Level II and Level III responses may also have adverse effects on PTB risk. Therefore, we quantitatively estimated individual cumulative exposure dose to lockdown by assigning different weightings to days with different levels of emergency responses: 1/22/2020 or earlier (no response, weighting = 0), 1/23–2/24/2020 (Level I, weighting = 3), 2/25–5/9/2020 (Level II, weighting = 2), and 5/10–12/31/2020 (Level III, weighting = 1). Moreover, to account for the potential effect modification by the timing of exposure, we only estimated the cumulative exposure dose in their first 22 GWs, a conventional cut-off value of the shortest GW for a newborn to survive with current medical technology (Fig. 2) [29]. The distribution of the lockdown exposure dose in the exposed group is shown in Fig. S4.

### Outcome measures

According to the World Health Organization, PTB was defined as gestational length ≤ 37 completed weeks [30]. Moderate PTB (MPTB) was defined as the gestational length between 32 and 36 completed weeks. Very PTB (VPTB) was defined as gestational length < 32 completed weeks. The VPTB included extremely PTB (gestational length < 28 completed weeks).

### Potential confounders

The following variables were considered as potential confounders: maternal age, marital status, parity, residential district, delivery type, and infant sex. These variables were selected based on biological plausibility, literature review, and availability of information.

### Potential mediators

To facilitate interpretation of our findings regarding the association between COVID lockdown and PTB, we considered pregnancy complications (HDP and GDM) and changes in air pollution around lockdown beyond regular seasonal variation as two potential mediators. HDP included gestational hypertension, preeclampsia/eclampsia, chronic hypertension, and chronic hypertension with superimposed preeclampsia [31, 32] (Table S2). Daily air pollutants (PM_10_, PM_2.5_, NO_2_, SO_2_, and CO) data in the selected cities during 2015–2020 were collected from the National Urban Air Quality Real-time Publishing Platform (http://106.37.208.233:20035/). The average air pollutant concentrations during, after the Level I lockdown in 2020, and during the same calendar months in 2015–2019 were calculated.

### Statistical analysis

A Chi-square test was used to assess the differences in socio-demographic and pregnancy characteristics between the exposed and unexposed groups. A generalized linear model (GLM) was applied to estimate the associations of Level I lockdown exposure with gestational length (linear regression) and PTB risk (binary logistic regression), after adjusting for potential confounders. A multinomial logistic regression model was used when PTB was further divided into MPTB and VPTB, with term birth as the reference. An interaction test was conducted to examine the potential modification effects of infant sex by comparing the association coefficients between male and female infants [33].

Similarly, GLM and multinomial logistic regression models were employed to examine the association of cumulative exposure dose with gestational length or PTB. The cumulative exposure dose in the exposed group was divided into four groups by quartiles (Q1, Q2, Q3, and Q4). The association of each quartile of cumulative exposure (vs unexposed) with gestational length or PTB was estimated. A trend test was conducted by assuming the values of quartiles as a continuous variable.

All analyses were performed using R3.6.1 (R Development Core Team 2019, https://www.r-project.org). The map in Fig. 1 was performed using packages *ggplot2* in the R3.6.1. All the tests were two-sided and a *P* <  0.05 was statistically significant.

## Results

### General characteristics of study participants

Out of the 595,396 women included, 101,900 (17.1%) were in the exposed group and the other 493,496 (82.9%) were in the unexposed group (Table 1). The exposed group had higher proportions of participants older than 30 years (52.8% vs 49.4%), with GDM (15.4% vs 12.3%), multiparity (21.8% vs 15.9%), and natural delivery (62.2% vs 60.3%), but a lower proportion of HDP (2.3% vs 2.7%) than the unexposed group.

**Table 1:** General characteristics of study participants

	**Unexposed group (*n*** **= 493,496)** **No. of participants (%)**	**Exposed group (*n*** **= 101,900)** **No. of participants0 (%)**	***χ***^**2**^	***P***
**Maternal age (years)**				
< 24	50,255 (10.2)	8412 (8.3)	660.24	< 0.001
24–26	81,222 (16.5)	15,864 (15.6)		
27–29	118,040 (23.9)	23,723 (23.3)		
30–32	102,817 (20.8)	23,585 (23.1)		
33–35	72,330 (14.7)	16,094 (15.8)		
> 35	68,832 (13.9)	14,222 (13.9)		
**Residential city**				
Guangzhou	19,850 (4.0)	2970 (2.9)	1193.80	< 0.001
Dongguan	34,579 (7.0)	5641 (5.5)		
Jiangmen	18,107 (3.7)	3303 (3.3)		
Shenzhen	75,334 (15.3)	13,280 (13.0)		
Foshan	345,626 (70.0)	76,706 (75.3)		
**Infant sex**			< 0.01	0.950
Male	263,153 (53.3)	54,349 (53.3)		
Female	230,343 (46.7)	47,551 (46.7)		
**Pregnancy complications (*N*** **= 173,064)**^**a**^				
*Hypertensive disorders of pregnancy (HDP)*				
No	143,933 (97.3)	24,369 (96.7)	96.57	< 0.001
Yes	3937 (2.7)	825 (2.3)		
Gestational hypertension	971 (0.7)	252 (1.0)		
Pre-eclampsia / Eclampsia	2712 (1.8)	473 (1.9)		
Chronic hypertension	141 (0.1)	44 (0.2)		
Chronic hypertension with superimposed pre-eclampsia	113 (0.1)	56 (0.2)		
*Gestational diabetes mellitus (GDM)*				
No	129,653 (87.7)	21,313 (84.6)	183.64	< 0.001
Yes	18,217 (12.3)	3881 (15.4)		
**Preterm birth**				
No	467,865 (94.8)	96,307 (94.5)	15.58	< 0.001
Yes	25,631 (5.2)	5593 (5.5)		
Very premature (< 32 GWs)	2121 (0.4)	443 (0.4)		
Moderate/late premature (32–36 GWs)	23,510 (4.8)	5150 (5.1)		
**Stillbirth (*N*** **= 595,904)**				
No	493,496 (99.92)	101,900 (99.90)	3.36	0.067
Yes	405 (0.08)	103 (0.10)		
**Marital status**				
Married	488,376 (99.0)	100,631 (98.8)	472.03	< 0.001
Unmarried	4263 (0.8)	732 (0.7)		
Other	857 (0.2)	537 (0.5)		
**Parity**				
0 (Primiparas)	415,074 (84.1)	79,686 (78.2)	2121.60	< 0.001
1 (Multiparas)	63,158 (12.8)	17,603 (17.3)		
2–4 (Multiparas)	15,264 (3.1)	4611 (4.5)		
**Delivery type**				
Natural delivery	297,591 (60.3)	63,394 (62.2)	1871.40	< 0.001
Operative vaginal delivery	16,735 (3.4)	1055 (1.0)		
Cesarean delivery	179,027 (36.3)	37,298 (36.6)		
Other	143 (< 0.1)	153 (0.2)		
	**Mean ± SD**	**Mean ± SD**	***t***	***P***
**Maternal age (years)**	29.78 ± 5.09	30.07 ± 4.94	17.11	< 0.001
**Gestational length (week Mean ± SD)**	38.74 ± 1.46	38.66 ± 1.46	16.22	< 0.001

^a^ Data that were not available in hospitals in Foshan, because the information were not recorded in the birth certification system

### Associations of COVID-19 lockdown exposure with gestational length

The exposed group had a shorter gestational length than the unexposed group (38.66 ± 1.46 weeks vs 38.74 ± 1.46 weeks). The Level I response (vs no exposure) was significantly associated with a 0.06 (95%CI: 0.05, 0.07) week decrease in gestational length in the total study sample after adjusting for confounders (Table 2). Subgroup analyses showed significant associations between lockdown exposure and decreased gestational length only among pregnant women whose gestational ages were < 24 GWs or 28th-31st GWs on the first day of lockdown (1/23/2020). The mean difference varied between − 0.11 and − 0.04 weeks.

**Table 2:** Associations of exposure to the COVID-19 lockdown with gestational length

	**No. of participants**		**Gestational length (week, Mean ± SD)**		**Mean difference in gestational length (week)**	
	**Unexposed group**	**Exposed group** ^**b**^	**Unexposed group**	**Exposed group** ^**b**^	**Crude** ***β*** **(95% CI)**	**Adjusted** ***β*** **(95% CI)** ^*^
**Gestational week at the beginning of the Level I lockdown**						
All	493,496	101,900	38.74 ± 1.46	38.66 ± 1.46	−0.08 (−0.09, −0.07)	−0.06 (−0.07, −0.05)
Conception during the lockdown	64,645	11,317	38.72 ± 1.52	38.64 ± 1.49	−0.08 (− 0.11, − 0.05)	−0.04 (− 0.07, − 0.01)
Prior to 4th	53,300	10,937	38.71 ± 1.50	38.64 ± 1.50	−0.07 (− 0.10, − 0.04)	− 0.10 (− 0.14, − 0.07)
4th -7th	50,973	10,494	38.67 ± 1.52	38.52 ± 1.54	− 0.14 (− 0.17, − 0.11)	− 0.13 (− 0.16, − 0.09)
8th -11th	48,926	10,237	38.70 ± 1.50	38.58 ± 1.54	−0.12 (− 0.15, − 0.08)	−0.10 (− 0.13, − 0.07)
12th -15th	46,255	9844	38.73 ± 1.51	38.61 ± 1.55	−0.11 (− 0.15, − 0.08)	−0.11 (− 0.14, − 0.07)
16th -19th	45,913	9539	38.74 ± 1.48	38.63 ± 1.52	−0.11 (− 0.14, − 0.08)	−0.10 (− 0.13, − 0.06)
20th -23rd	41,017	8830	38.74 ± 1.49	38.64 ± 1.52	−0.10 (− 0.14, − 0.07)	−0.10 (− 0.13, − 0.06)
24th -27th	40,358	8750	38.68 ± 1.49	38.66 ± 1.44	−0.02 (− 0.06, 0.01)	−0.01 (− 0.04, 0.03)
28th -31st	38,146	8101	38.72 ± 1.39	38.63 ± 1.36	−0.09 (− 0.12, − 0.06)	−0.07 (− 0.10, − 0.04)
32nd -36th	47,382	10,213	38.74 ± 1.21	38.73 ± 1.18	−0.01 (− 0.03, 0.02)	0.02 (− 0.01, 0.04)
37th - 41st	16,581	3638	39.40 ± 0.92	39.41 ± 0.93	0.01 (−0.02, 0.04)	0.03 (0.01, 0.07)
	**Exposure dose (Mean ± SD)**		**Gestational length (week, Mean ± SD)**		**Mean difference in gestational length (week)**	
	**Unexposed group**	**Exposed group**	**Unexposed group**	**Exposed group**	**Crude** ***β*** **(95% CI)**	**Adjusted** ***β*** **(95% CI)** ^*^
**Cumulative exposure dose in the first 22 weeks during the Level I to the Level III lockdown** ^**a**^						
Per 100 unit increase in all participants	0 ± 0	195.08 ± 82.21	38.74 ± 1.45	38.61 ± 1.52	−0.06 (−0.07, −0.05)	−0.05 (− 0.06, − 0.04)
**Categories of cumulative exposure dose**						
Unexposed group	0 ± 0	–	38.74 ± 1.45	–	Reference	Reference
Q_1_ (< 132)	–	73.40 ± 38.11	–	38.64 ± 1.52	−0.10 (− 0.13, − 0.08)	−0.09 (− 0.11, − 0.07)
Q_2_ (132–225)	–	178.66 ± 27.17	–	38.59 ± 1.54	−0.15 (− 0.17, − 0.12)	−0.13 (− 0.16, − 0.11)
Q_3_ (226–263)	–	247.18 ± 10.58	–	38.58 ± 1.51	−0.16 (− 0.18, − 0.13)	−0.14 (− 0.16, − 0.11)
Q_4_ (≥264)	–	278.80 ± 8.59	–	38.62 ± 1.51	− 0.12 (− 0.14, − 0.10)^*^	−0.09 (− 0.11, − 0.07)
*P* for trend test						< 0.001

In calculating the cumulative exposure dose to lockdown, we assigned a weighting of 3 to days with Level I response, 2 to days with Level II response, 1 to days with Level III response, and 0 to other days

* Adjusted for maternal age, marital status, parity, residential city, delivery type and infant sex

^a^ The exposed group refers to the pregnant women who have experienced the COVID-19 lockdown in their first 22 GWs. The other participants were defined as the unexposed group. The individual cumulative exposure dose was calculated by combining the weightings with the overlap between their pregnancy period ≤22 GWs and the three levels of responses. Q_1_-Q_4_ were defined as the cumulative exposure dose of the exposed group classified by quartiles, and the unexposed group was used as reference

^b^ Pregnant women who have experienced the COVID-19 lockdown (from 1/23/2020 to 2/24/2020) during any period of their pregnancy were defined as the exposed group. We further divided the exposed group into subgroups according to their gestational weeks (GW) on 1/23/2020, the beginning of lockdown

-: Not applicable

We observed a negative association between cumulative lockdown exposure dose and gestational length (Table 2). Each 100 unit increase in the cumulative exposure dose during the first 22 GWs was associated with a 0.05 (95%CI: 0.04, 0.06) week decrease in gestational length, after adjusting for confounders. In addition, compared to the unexposed group, the Q1, Q2, Q3 and Q4 quantiles of cumulative exposure were associated with 0.09 (0.07, 0.11), 0.13 (0.11, 0.16), 0.14 (0.11, 0.16), and 0.09 (0.07, 0.11) weeks decrease in gestational length, respectively.

### Associations of COVID-19 lockdown exposure with PTB

A higher PTB rate (5.7% vs 5.3%) and MPTB rate (5.2% vs 4.9%) were observed in the exposed group compared to the unexposed group in the total sample. Significant increases in PTB risk (adjusted OR = 1.08, 95%CI: 1.05, 1.11) and MPTB risk (adjusted OR = 1.09, 95%CI: 1.05, 1.12) were also observed after adjusting for confounders (Table 3). However, the association between lockdown and VPTB was not statistically significant (adjusted OR = 1.04, 95%CI: 0.94, 1.16). Subgroup analyses showed significant associations of lockdown exposure with increases in PTB and MPTB only among pregnant women < 24 GWs on the first day of lockdown. The OR values varied between 1.10 and 1.20 for PTB and MPTB.

**Table 3:** Associations of exposure to the COVID-19 lockdown with preterm birth

	**Unexposed group (n, %)**				**Exposed group (n, %)** ^**a**^				**OR for PTB (95%CI)**					
	**Term birth**	**PTB**			**Term birth**	**PTB**			**MPTB + VPTB**		**MPTB**		**VPTB**	
		**MPTB + VPTB**	**MPTB**	**VPTB**		**MPTB + VPTB**	**MPTB**	**VPTB**	**Crude OR**	**Adjusted OR** ^*^	**Crude OR**	**Adjusted OR** ^*^	**Crude OR**	**Adjusted OR** ^*^
**Gestational week at the beginning of the Level I lockdown**														
All	451,284 (94.7)	25,631 (5.3)	23,510 (4.9)	2121 (0.4)	92,669 (94.3)	5593 (5.7)	5150 (5.2)	443 (0.5)	1.06 (1.03, 1.09)	1.08 (1.05, 1.11)	1.07 (1.03, 1.10)	1.09 (1.05, 1.12)	1.02 (0.92, 1.13)	1.04 (0.94, 1.16)
Conception during the lockdown	61,117 (94.5)	3528 (5.5)	3171 (4.9)	357 (0.6)	10,682 (94.4)	635 (5.6)	573 (5.1)	62 (0.5)	1.03 (0.94, 1.12)	1.09 (0.99, 1.20)	1.03 (0.94, 1.13)	1.08 (0.98, 1.19)	0.99 (0.76, 1.30)	1.20 (0.90, 1.61)
Prior to 4th	50,272 (94.3)	3028 (5.7)	2746 (5.2)	282 (0.5)	10,295 (94.1)	642 (5.9)	591 (5.4)	51 (0.5)	1.04 (0.95, 1.13)	1.18 (1.08, 1.29)	1.05 (0.96, 1.15)	1.20 (1.09, 1.32)	0.88 (0.65, 1.19)	0.97 (0.71, 1.32)
4th -7th	48,023 (94.3)	2950 (5.7)	2671 (5.2)	279 (0.5)	9821 (93.6)	673 (6.4)	613 (5.8)	60 (0.6)	1.12 (1.02, 1.22)	1.12 (1.03, 1.22)	1.12 (1.03, 1.23)	1.13 (1.03, 1.24)	1.05 (0.79, 1.39)	1.07 (0.81, 1.42)
8th -11th	46,128 (94.3)	2798 (5.7)	2544 (5.2)	254 (0.5)	9581 (93.6)	656 (6.4)	597 (5.8)	59 (0.6)	1.13 (1.03, 1.23)	1.14 (1.04, 1.24)	1.13 (1.03, 1.24)	1.14 (1.04, 1.25)	1.12 (0.84, 1.49)	1.13 (0.85, 1.51)
12th -15th	43,652 (94.4)	2603 (5.6)	2356 (5.1)	247 (0.5)	9236 (93.9)	608 (6.1)	546 (5.5)	62 (0.6)	1.10 (1.01, 1.21)	1.11 (1.02, 1.22)	1.10 (1.00, 1.21)	1.10 (1.00, 1.21)	1.19 (0.90, 1.57)	1.21 (0.91, 1.59)
16th -19th	43,439 (94.6)	2474 (5.4)	2252 (4.9)	222 (0.5)	8966 (94.0)	573 (6.0)	524 (5.5)	49 (0.5)	1.12 (1.02, 1.23)	1.14 (1.04, 1.25)	1.13 (1.02, 1.24)	1.14 (1.04, 1.26)	1.07 (0.78, 1.46)	1.11 (0.82, 1.52)
20th -23rd	38,806 (94.6)	2211 (5.4)	2011 (4.9)	200 (0.5)	8281 (93.8)	549 (6.2)	502 (5.7)	47 (0.5)	1.16 (1.06, 1.28)	1.19 (1.08, 1.31)	1.17 (1.06, 1.29)	1.19 (1.08, 1.32)	1.10 (0.80, 1.51)	1.14 (0.83, 1.57)
24th -27th	38,052 (94.3)	2306 (5.7)	2112 (5.2)	194 (0.5)	8291 (94.8)	459 (5.2)	420 (4.8)	39 (0.4)	0.91 (0.82, 1.01)	0.93 (0.84, 1.03)	0.91 (0.82, 1.02)	0.93 (0.83, 1.03)	0.92 (0.65, 1.30)	0.99 (0.70, 1.40)
28th -31st	36,145 (94.8)	2001 (5.2)	1915 (5.0)	86 (0.2)	7663 (94.6)	438 (5.4)	424 (5.2)	14 (0.2)	1.03 (0.93, 1.15)	1.05 (0.95, 1.17)	1.04 (0.94, 1.16)	1.06 (0.95, 1.19)	0.77 (0.44, 1.35)	0.80 (0.45, 1.42)
32nd -36th	45,650 (96.3)	1732 (3.7)	1732 (3.7)	NA	9853 (96.5)	360 (3.5)	360 (3.5)	NA	0.96 (0.86, 1.08)	0.97 (0.86, 1.09)	0.96 (0.86, 1.08)	0.97 (0.86, 1.09)	NA	NA
	**Exposure dose in unexposed group** **(Mean ± SD)**	**Exposure dose in exposed group** **(Mean ± SD)**					**OR for PTB (95%CI)**							
	**Term + PTB**	**Term**	**PTB**				**MPTB + VPTB**			**MPTB**		**VPTB**		
			**MPTB + VPTB**		**MPTB**	**VPTB**	**Crude OR**		Adjusted OR^*^	**Crude OR**	Adjusted OR^*^	**Crude OR**	Adjusted OR^*^	
**Cumulative exposure dose in the first 22 weeks during Level I to Level 3 lockdown** ^**b**^														
Per 100 unit increase	0 ± 0	195.14 ± 82.19	194.15 ± 82.57		194.23 ± 82.60	193.35 ± 82.38	1.06 (1.04, 1.08)		1.07 (1.05, 1.09)	1.05 (1.04 1.07)	1.07 (1.05, 1.08)	1.10 (1.05, 1.16)	1.12 (1.06, 1.18)	
**Categories of cumulative exposure dose**														
Unexposed group	0 ± 0	–					Reference		Reference	Reference	Reference	Reference	Reference	
Q_1_ (< 132)	–	73.52 ± 38.09	71.59 ± 38.28		71.49 ± 38.32	72.57 ± 38.07	1.14 (1.07, 1.22)		1.16 (1.08, 1.23)	1.13 (1.05, 1.21)	1.14 (1.07, 1.22)	1.27 (1.03, 1.67)	1.31 (1.06, 1.62)	
Q_2_ (132–225)	–	178.64 ± 27.17	179.01 ± 27.26		179.14 ± 27.26	177.76 ± 27.29	1.21 (1.13, 1.29)		1.22 (1.14, 1.30)	1.19 (1.11, 1.27)	1.20 (1.12, 1.28)	1.39 (1.14, 1.70)	1.43 (1.17, 1.74)	
Q_3_ (226–263)	–	247.16 ± 10.59	247.52 ± 10.28		247.50 ± 10.26	247.91 ± 10.58	1.11 (1.04 1.19)		1.14 (1.07, 1.22)	1.10 (1.03, 1.18)	1.13 (1.05, 1.21)	1.25 (1.01, 1.54)	1.27 (1.03, 1.58)	
Q_4_ (≥264)	–	278.81 ± 8.57	278.69 ± 8.77		278.55 ± 8.76	280.23 ± 8.79	1.14 (1.06, 1.21)		1.19 (1.11, 1.27)	1.13 (1.05, 1.21)	1.18 (1.10, 1.26)	1.21 (0.97, 1.49)	1.25 (1.01, 1.55)	
*P* for trend test									< 0.001		< 0.001		< 0.001	

In calculating the cumulative exposure dose to lockdown, we assigned a weighting of 3 to days with Level I response, 2 to days with Level II response, 1 to days with Level III response, and 0 to other days

*PTB* preterm birth, *MPTB* moderate preterm birth, *VPTB* very preterm birth

N/A: There is no VPTB case in the subgroup

*: Adjusted for maternal age, marital status, parity, residential city, delivery type and infant sex

^a^ Pregnant women who have experienced the COVID-19 lockdown (from 1/23/2020 to 2/24/2020) during any period of their pregnancy were defined as the exposed group. We further divided the exposed group into subgroups. According to their gestational weeks (GW) on 1/23/2020, the beginning of lockdown

b: The exposed group refers to the pregnant women who have experienced the COVID-19 lockdown in their first 22 GWs. The rest of included participants were defined as the unexposed group. The individual cumulative exposure dose was calculated by combining the weightings with the overlap between their pregnancy period ≤22 GWs and the three levels of responses. Q_1_-Q_4_ were defined as the cumulative exposure dose of the exposed group classified by quartiles, and the unexposed group were used as reference

-: Not applicable

We also observed a positive association between cumulative exposure dose to lockdown and PTB risk (Table 3 and Table S3). Each 100 unit increase in the lockdown exposure during the first 22 GWs was significantly associated with 1.07 (95%CI: 1.05, 1.09), 1.07 (1.05, 1.08), and 1.12 (1.06, 1.18) times higher risks in PTB, MPTB, and VPTB, respectively. The adjusted ORs of PTB for the Q1, Q2, Q3 and Q4 quartiles of cumulative exposure (vs no exposure) were 1.16 (1.08, 1.23), 1.22 (1.14, 1.30), 1.14 (1.07, 1.22), and 1.19 (1.11, 1.27), respectively.

### Effect modification by infant sex in the associations of lockdown exposure with gestational length and PTB

Subgroup analyses showed similar associations of Level I lockdown with gestational length [adjusted β = − 0.06 (95%CI: − 0.08, − 0.05) week vs adjusted β = − 0.06 (− 0.08, 0.05) week] or risk of PTB [adjusted OR = 1.09 (95%CI: 1.04, 1.14) vs adjusted OR = 1.08 (1.03, 1.13)] in male infants and in female infants (Table 4). There was no significant sex interaction (*P* >  0.05) in these associations.

**Table 4:** Modification effects of infant sex on the associations of COVID-19 lockdown exposure with gestational length and PTB risk

	**No. of participants**				**Gestational length (week, Mean ± SD)**				**Mean difference in gestational length (week)** **Adjusted** ***β*** **(95% CI)** ^*^		***P*** **for modification effects of infant sex**
	**Male**		**Female**		**Male**		**Female**		**Male**	**Female**	
	**Unexposed group**	**Exposed group** ^**a**^	**Unexposed group**	**Exposed group** ^**a**^	**Unexposed group**	**Exposed group** ^**a**^	**Unexposed group**	**Exposed group** ^**a**^			
**Gestational week during the Level I lockdown**											
All	263,153	54,349	230,343	47,551	38.66 ± 1.48	38.58 ± 1.48	38.82 ± 1.43	38.74 ± 1.44	−0.06 (−0.08, −0.05)	−0.06 (−0.08, −0.05)	> 0.05
	**No. of participants**				**PTB rate (N, %)**				**PTB risk** **Adjusted OR (95%CI)** ^*^		***P*** **for modification effects of infant sex**
	**Male**		**Female**		**Male**		**Female**		**Male**	**Female**	
	**Unexposed group**	**Exposed group** ^**a**^	**Unexposed group**	**Exposed group** ^**a**^	**Unexposed group**	**Exposed group** ^**a**^	**Unexposed group**	**Exposed group** ^**a**^			
**Gestational week during the Level I lockdown**											
All	254,522	52,471	222,393	45,791	14,873 (5.8)	3269 (6.2)	10,758 (4.8)	2324 (5.1)	1.09 (1.04, 1.13)	1.08 (1.03, 1.13)	> 0.05

*PTB* preterm birth

^*^ Adjusted for maternal age, marital status, residential city, delivery type and parity

^a^ Pregnant women who have experienced the COVID-19 lockdown (from 1/23/2020 to 2/24/2020) during their any period of pregnancy were defined as the exposed group

## Discussion

This study comprehensively examined the associations of the COVID-19 lockdown with gestational length and risk of PTB using a large database from South China. We found that the lockdown exposure was significantly associated with a slightly shorter gestational length and a moderately higher risk of PTB. These associations were greater among women who were in early or middle pregnancy during the Level I lockdown period. There were also significant exposure-response associations of higher cumulative exposures to lockdown with a shorter gestational length and an increased risk of PTB.

Our finding of a positive association between the COVID-19 lockdown and risk of PTB was consistent with some previous studies. For example, a study from California found a modest increase in PTB rates (OR = 1.11; 95% CI, 1.03–1.20) among pregnant women at 28 + 0 and 31 + 6 weeks during the COVID-19 pandemic compared with 2016–2019 [34]. Another study from Italy also reported a slight increase in very preterm rate during the COVID-19 lockdown period in 2020 (0.79%) compared with the same period in 2019 (0.55%) [35]. Several reasons possibly explained the increased risk of PTB. First, the lack of medical resources during the COVID-19 pandemic and lockdown measures might interrupt the timely antenatal care for pregnant women [13, 36, 37]. Secondly, fear and panic about the pandemic could make pregnant women reluctant to seek help from medical institutions, and further impacted the timely detection and diagnosis of pregnancy complications [13, 38]. For example, we observed a higher rate of GDM in the exposed group than the unexposed group. This suggested a potential mediation role of GDM, as GDM is a critical risk factor of PTB [14]. In addition, pregnant women have always been considered a susceptible population to mental disorders [39]. The lockdown and restriction measures could increase psychological problems in pregnant women through concomitant financial problems and increased stress [38, 40], particularly if they were socioeconomically disadvantaged [41]. The closure of entertainment venues also reduced the outlets for negative feelings [42]. A previous study observed a more pronounced increase in depression and anxiety in pregnant women during the COVID-19 pandemic than in the general population [43]. Lastly, the nutritional status of pregnant women was also of concern. During the lockdown period, the decreased supply of fresh foods could lead to an inadequate intake of vegetables and high-fiber foods. Meanwhile, the intake of high-carbohydrate foods might have increased because they were relatively easier to obtain and store [13]. It was reported that the overweight and obesity rates increased during the lockdown period due to unbalanced diets and less exercise [44]. This suggested that maternal stress and obesity during the lockdown might influence the risk of PTB [13, 43].

We further observed that women in early and middle pregnancy during the Level I lockdown had a greater risk of PTB, which also contributed to the health effects of the COVID-19 lockdown. Zhang et al. reported that women in the first and second trimesters of pregnancy during the lockdown had more severe psychological disorders [22]. A simple explanation could be that these mothers continued to experience Level II and III lockdown after the Level I lockdown, which may have led to more cumulative effects on their fetal health. This was supported by our observed positive association between PTB risk and cumulative exposure to the lockdown of all levels in the first 24 GWs. An alternative explanation could be that early and middle pregnancy is a critical period for fetal development because the majority of fetal organs and tissues retain plasticity at that time [45]. As a result, lockdown-induced poor diet, depression, and anxiety problems in early and middle pregnancy may substantially interrupt fetal development [46–48].

It should be noted that several other previous studies reported different results with our findings. For example, studies in Denmark and the Netherlands have both provided evidence of a significant reduction in the number of extremely preterm and a decline in the incidence of moderate-to-late preterm birth following the implementation of national COVID-19 mitigation measures compared with years prior to the COVID-19 pandemic [16, 17]. A large sample study in China also found a statistically significant decrease in the incidence of moderate-to-late preterm birth during the COVID-19 mitigation measures compared with the same periods during 2014–2019 [19]. However, studies in the US states of Pennsylvania and Massachusetts found no change in the lockdown-related PTB during the COVID-19 pandemic [49, 50]. Although the mechanisms underlying these negative or null associations were unclear, several socio-environmental and behavioral modifiers were proposed [11, 51]. First, the lockdown measures increased company and support from partners and family, which could reduce the existing psychological stress in pregnant women. Second, working from home increased their rest time at home and decreased work-related stress. Third, the reduced anthropogenic emissions improved the air quality, which could benefit maternal and fetal health. Fourth, precautionary behavioral changes were promoted during the lockdown, including social distancing, enhanced hand hygiene, and the use of face masks. These behavioral changes could potentially reduce the chances of other common viral infections in addition to COVID-19 during pregnancy. Finally, lockdown measures also reduced daily commuting, road traffic incidents, and consumption of cigarettes, coffee, alcohol, prescription drugs, and street drugs due to limited accessibility [11, 51].

Those inconsistent associations of lockdown exposure with maternal and fetal health reported in previous studies [11, 16, 17] may have a few other explanations. First, some studies [16] had small sample sizes and potentially inadequate statistical power to detect an association between lockdown exposure and PTB. Second, the seemingly decreased risk of adverse pregnancy outcomes related to lockdown might be partially related to the reduced number of ultrasound scans and screening, which increased the possibility of under-diagnoses of early pregnancy loss, miscarriages, or stillbirths. Third, the health effects of lockdown may last for several months, but previous studies did not track participants long enough to assess the total effects of lockdown, which could have led to underestimations. In this study, we used the data of pregnant women who experienced the Level I lockdown until the end of 2020 and were able to obtain birth outcomes of all exposed women by covering the entire pregnancy. Fourth, air quality improvement during the lockdown was proposed as a major contributor to the reduced risk of PTB. In this study, we also found a substantial reduction in air pollution during the lockdown (Table S4), which was consistent with previous studies [6, 52]. Fifth, seasonal effects and pregnancy stages were not considered in most previous studies, which could lead to biased results. To evaluate this potential bias, we estimated the difference in PTB rates between new births during the Level I lockdown and all previous births during the entire year (rather than matching the calendar months) from 2015 to 2019. We did not find a significant association between lockdown and PTB risk (Table S5). Finally, although the lockdown measures may increase company and support from partners and family, the potential increase in family conflicts and domestic abuse should also be considered [53]. These findings suggest that the health effects of COVID-19 lockdown were comprehensively affected by socio-environmental changes and behavioral modifications and that improvement in one factor could not make up for the overall disadvantage [16, 34].

### Limitations

Several limitations need to be addressed. First, as the COVID-19 pandemic and associated lockdown measures occurred unanticipatedly, we can only extract general demographic information, pregnancy complications and pregnancy outcomes from hospital information systems or birth record systems. We were unable to gather information on the management of high-risk preterm pregnancies, such as the frequency and form of examination of these patients, gynecological examination, serology, bacteriological findings of cervical and vaginal swabs, ultrasound examination of cervical length and some others. In addition, we may miss some other gestation-length-related outcomes such as early pregnancy losses, miscarriages, and stillbirths. These competing outcomes of PTB may potentially downplay the impact of lockdown on pregnancy and PTB [19]. Previous studies reported an increased rate of stillbirth related to the COVID-19 lockdown [12, 15]. Our supplemental analyses also showed a higher stillbirth risk in the exposed group than in the unexposed group (Table S6). Second, some studies have shown that smoking, alcohol consumption and reduced physical activity during pregnancy can lead to higher rates of PTB [54–56]. But we can’t get the individual behavior of these pregnant women from the hospital system, so their potential mediation roles were not evaluated in our analyses. Third, this study was conducted in only five cities in South China, which limited the generalization of our findings. However, we chose representative hospitals in each city. For example, Shenzhen Maternal and Child Health Hospital is the largest local hospital which provided medical serves to people across the city. Fourth, due to the coexistence of the COVID-19 pandemic and lockdown status, we could not separate their induvial impacts on the outcomes. In addition, many measures are usually implemented at the same time, we could not also determine their impact on the risk of PTB.

## Conclusions

Within a large dataset of birth records from South China, we found that COVID-19 lockdown was associated with a slightly shorter gestational length and a moderately higher risk of PTB. Early and middle pregnancy might be a more susceptible exposure window. The COVID-19 control measures were implemented in many countries to reduce the spread of infections and related morbidities. Meanwhile, the incidence of PTB remains high globally, and options for the prevention of PTB are very limited [57]. Our findings suggest more attention and efforts are needed to support pregnant women during the lockdown, particularly for those with previous PTB as they are more susceptible [58]. Health professionals should make appropriate and timely treatment decisions for pregnant women during the lockdown.

## Supplementary Information

**Additional file 1 **: **Table S1**. The prevention and control measures of three level response in Guangdong province. **Table S2**. Definitions of pregnancy complications selected in this study. **Table S3**. Proportions of study participants in the exposed and unexposed groups. **Table S4**. The differences in air pollution between lockdown and pre-lockdown periods in the study area. **Table S5**. Associations between exposure to COVID-19 lockdown at birth and preterm birth. **Table S6**. Associations of exposure to COVID-19 lockdown with stillbirth. **Figure S1**. Selection process of study subjects. **Figure S2**. Rate of preterm birth in each calendar month during 2015-2019 (before the COVID-19 pandemic). **Figure S3**. Division of participants into subgroups according to their GW on January 23rd, 2020. **Figure S4**. Distribution of the cumulative exposure dose in the first 22 GWs in the exposed group during lockdown.

## Data Availability

The statistical code and meta data during the current study are not publicly available due to the qualitative nature of the data, but are available from the corresponding author upon request.

## Abbreviations

COVID-19: Coronavirus disease 2019
PTB: Preterm birth
MPTB: Moderate preterm birth
VPTB: Very preterm birth
GW: Gestational week
HDP: Hypertensive disorders of pregnancy
GDM: Gestational diabetes mellitus.
