# Supplementary material for: Associations of COVID-19 lockdown with gestational length and preterm birth in China
Source: BMC Pregnancy Childbirth. 2021 Nov 27;21:795. doi: 10.1186/s12884-021-04268-5 (PMC8626761; doi:10.1186/s12884-021-04268-5)
Supplement: Supplementary file 1 — Additional file 1 : Table S1. The prevention and control measures of three level response in Guangdong province. Table S2. Definitions of pregnancy complications selected in this study. Table S3. Proportions of study participants in the exposed and unexposed groups. Table S4. The differences in air pollution between lockdown and pre-lockdown periods in the study area. Table S5. Associations between exposure to COVID-19 lockdown at birth and preterm birth. Table S6. Associations of exposure to COVID-19 lockdown with stillbirth. Figure S1. Selection process of study subjects. Figure S2. Rate of preterm birth in each calendar month during 2015-2019 (before the COVID-19 pandemic). Figure S3. Division of participants into subgroups according to their GW on January 23rd, 2020. Figure S4. Distribution of the cumulative exposure dose in the first 22 GWs in the exposed group during lockdown. [file 12884_2021_4268_MOESM1_ESM.doc]

**Supplementary Meterial**

In order to compare with previous studies, we conducted a sensitivity analysis by using the same approach to estimate the association of COVID-19 lockdown with PTB. We defined all births during the level I lockdown period as the exposed group, and all births during 2015-2019 as the unexposed group. An unconditional logistic regression model was used to estimate the associations of lockdown exposure with PTB risk. A multinomial logistic regression model was used when PTB was further divided into MPTB and VPTB (Table S5).

**Supplementary tables**

**Table S1. The prevention and control measures of three level response in Guangdong province.**

| **The levels of emergency response** | **Prevention and control measures** | **Weighting**  **score** |
| --- | --- | --- |
| **Level I response** | - Body temperature monitoring and information registration implemented at the entrance of communities and opened public places like shops. Traffic quarantine inspection strictly implemented at airports, wharves, and inter-provincial bus stations. Cleaning and sanitation of key places such as markets to eliminate the possible breeding places for the virus. - Residents’ social movement and gathering rigorously restricted. Masks mandated to be worn in public transportation like buses, taxis, trains, and all crowded public places. - Strong management of COVID-19 patients, suspected patients, and close contacts. The "four centralized" prevention, control, and treatment measures adopted: centralized patients, centralized experts, centralized resources, and centralized treatment. Symptom surveillance was strictly carried out of people who entered Guangdong Province from other places. Medical supplies and safety equipment ensured, such as ambulances, disinfectants, testing reagents, medical devices, and protective equipment. The production and allocation of urgently needed materials promoted. | 3 |
| **Level II response** | - Some public places (i.e. public transportation, grocery stores, and gas station) and residential communities reopened. Shopping malls, cinemas, internet cafes, and other public places gradually opened up. Body temperature monitoring was continued to be implemented, and people required to maintain social distancing. - Masks required in public transportation (i.e. buses, taxis, trains) and in all crowded public places, but no need to wear a mask in an open area. - Buildings with confirmed or suspected cases closed and quarantined. | 2 |
| **Level III response** | - Social activities, commercial actives, and schools were gradually returned be normal. - All prevention and control measures transferred to new-normal status, including mask-wearing, keeping social distance, and body temperature monitoring in public places. - Symptom surveillance in health care institutes. | 1 |
| **Prior to COVID-19** | - No lockdown measures. | 0 |

**Table S2 Definitions of pregnancy complications selected in this study**

| Pregnancy complications | Definitions | References |
| --- | --- | --- |
| Gestational hypertension | Maternal blood pressure (BP) ≥ 140/90 mmHg on two separate occasions at least 4 hours apart, without proteinuria after 20 weeks of gestation, which returns to normal within 42 days postpartum. | 1 |
| Preeclampsia | Maternal BP ≥ 140/90 mmHg, with two readings taken at least 15 min apart with proteinuria after 20 weeks of gestation | 1 |
| Eclampsia | Eclampsia is tonic-clonic convulsions in women with preeclampsia. | 1 |
| Chronic hypertension | Maternal BP ≥ 140/90 mmHg before 20 weeks of pregnancy. | 1 |
| Superimposed preeclampsia | A new occurrence of preeclampsia in pregnant patients with chronic hypertension. | 1 |
| Gestational diabetes mellitus (GDM) | At 24-28 weeks of gestation using a 75 g oral glucose tolerance test (OGTT) when pregnant women satisfied either one of the following conditions: fasting blood glucose ≥5.1 mmol/L, 1 h blood glucose ≥ 10.0 mmol/L, and 2 h blood glucose ≥ 8.5 mmol/L. | 2 |

**Table S3. Proportions of study participants in the exposed and unexposed groups**

|  | **No. of participants (%)** | | | |
| --- | --- | --- | --- | --- |
|  | **Term** | **PTB** | | |
|  |  | **MPTB+VPTB** | **MPTB** | **VPTB** |
| **Cumulative exposure dose** **in the first 22 weeks during the Level 1 to the Level 3 lockdown ^a^** |  |  |  |  |
| Unexposed group | 481148 (94.7) | 27151 (5.3) | 24954 (4.9) | 2197 (0.4) |
| Exposed group divided by quartiles | 62805 (93.9) | 4073 (6.1) | 3706 (5.5) | 367 (0.6) |
| Q_1_ (<134) | 15479 (93.9) | 997 (6.1) | 907 (5.5) | 90 (0.6) |
| Q_2_ (134 - 228) | 15841 (93.6) | 1079 (6.4) | 978 (5.8) | 101 (0.6) |
| Q_3_ (228 - 265) | 15483 (94.1) | 972 (5.9) | 884 (5.4) | 88 (0.5) |
| Q_4_ (≥265) | 16002 (94.0) | 1025 (6.0) | 937 (5.5) | 88 (0.5) |

PTB: preterm birth; MPTB: moderate preterm birth; VPTB: very preterm birth

To calculate the cumulative exposure dose to lockdown, we assigned a weighting of 3 to days with Level I response, 2 to days with Level II response, 1 to days with Level III response, and 0 to other days.

a: The exposed group refers to the pregnant women who have experienced the COVID-19 lockdown in their first 22 GWs. The rest of included participants were defined as the unexposed group. The individual cumulative exposure dose was calculated by combining the weightings with the overlap between their pregnancy period ≤22 GWs and the three levels of responses. Q1-Q4 were defined as the cumulative exposure dose of the exposed group classified by quartiles.

**Table S4. The differences in air pollution between lockdown and pre-lockdown periods in the study area**

|  | **Average air pollutant concentrations (Mean ± SD)** | | | |
| --- | --- | --- | --- | --- |
|  | **During level I COVID-19 lockdown periods**  **(1/23/2020-2/24/2020)** | **The same calendar months (1/23-2/24) of 2015-2019 as the lockdown duration in 2020** | **Level II - III COVID-19 lockdown periods**  **(2/25/2020-12/31/2020)** | **The same calendar months (2/25-12/31) of 2015-2019 as the duration after lockdown in 2020** |
| **PM_10_ (μg/m^3^)** | 32.6 (20.0) | 63.1 (36.7) | 41.7 (28.3) | 54.2 (35.7) |
| **PM_2.5_ (μg/m^3^)** | 24.1 (15.8) | 42.4 (25.3) | 21.7 (16.2) | 31.9 (22.0) |
| **NO_2_ (μg/m^3^)** | 5.5 (1.8) | 9.2 (6.6) | 7.0 (3.2) | 10.0 (6.8) |
| **SO_2_ (μg/m^3^)** | 24.4 (19.5) | 46.2 (34.9) | 37.2 (24.4) | 47.1 (31.3) |
| **CO (mg/m^3^)** | 0.7 (0.2) | 0.9 (0.4) | 0.7 (0.2) | 0.8 (0.3) |

Note: air pollution data in the 5 selected cities during 2015-2020 was obtained from the National Urban Air Quality Real-time Publishing Platform (http://106.37.208.233:20035/). The average air pollutant concentrations during the lockdown (1/23/2020 to 2/24/2020) and after the lockdown (2/25/2020 to 12/31/2020) and the same calendar months in 2015-2019 were calculated.

**Table S5. Associations between exposure to COVID-19 lockdown at birth and preterm birth**

| **Unexposed group (n, %) ^a^** | | | |  | **Exposed group (n, %) ^b^** | | | |  | **OR for PTB (95%CI)** | | | | | |
| --- | --- | --- | --- | --- | --- | --- | --- | --- | --- | --- | --- | --- | --- | --- | --- |
| **Term birth** | **PTB** | | |  | **Term birth** | **PTB** | | |  | **MPTB+VPTB** | | **MPTB** | | **VPTB** | |
|  | **MPTB+VPTB** | **MPTB** | **VPTB** |  |  | **MPTB+VPTB** | **MPTB** | **VPTB** |  | **Crude OR** | **Adjusted OR ^*^** | **Crude OR** | **Adjusted OR ^*^** | **Crude OR** | **Adjusted OR ^*^** |
| 566687 (94.1) | 35304 (5.9) | 32032 (5.3) | 3272 (0.6) |  | 8661 (94.1) | 545 (5.9) | 501 (5.4) | 44 (0.5) |  | 1.01 (0.93, 1.10) | 1.01 (0.93, 1.11) | 1.02 (0.93, 1.12) | 1.03 (0.94, 1.12) | 0.88 (0.65, 1.19) | 1.04 (0.89, 1.20) |

*: Adjusted for maternal age, marital status, parity, residential city, delivery type and infant sex.

PTB: preterm birth; MPTB: moderate preterm birth; VPTB: very preterm birth

a: All births between 1/1/2015 to 1/22/2020 were defined as the unexposed group.

b: All births during the COVID-19 lockdown (1/23/2020 to 2/24/2020) were defined as the exposed group.

**Table S6. Associations of exposure to COVID-19 lockdown with stillbirth**

| **Unexposed group (n, %)** | | **Exposed group (n, %) ^a^** | | **OR for stillbirth (95%CI)** | |
| --- | --- | --- | --- | --- | --- |
| **Live birth** | **Stillbirth** | **Live birth** | **Stillbirth** | **Crude OR** | **Adjusted OR^*^** |
| 493496 (99.92) | 405 (0.08) | 101900 (99.90) | 103 (0.10) | 1.23 (0.99, 1.53) | 1.26 (1.01, 1.57) |

*: Adjusted for maternal age, marital status, parity, residential city, delivery type and infant sex.

a: Pregnant women who experienced the COVID-19 lockdown (from 1/23/2020 to 2/24/2020) during any period of their pregnancy were defined as the exposed group. Women whose pregnancy had an overlap with the same calendar months in 2015-2019 were defined as the unexposed group.

**Supplementary figures**

**Figure S1. Selection process of study subjects**


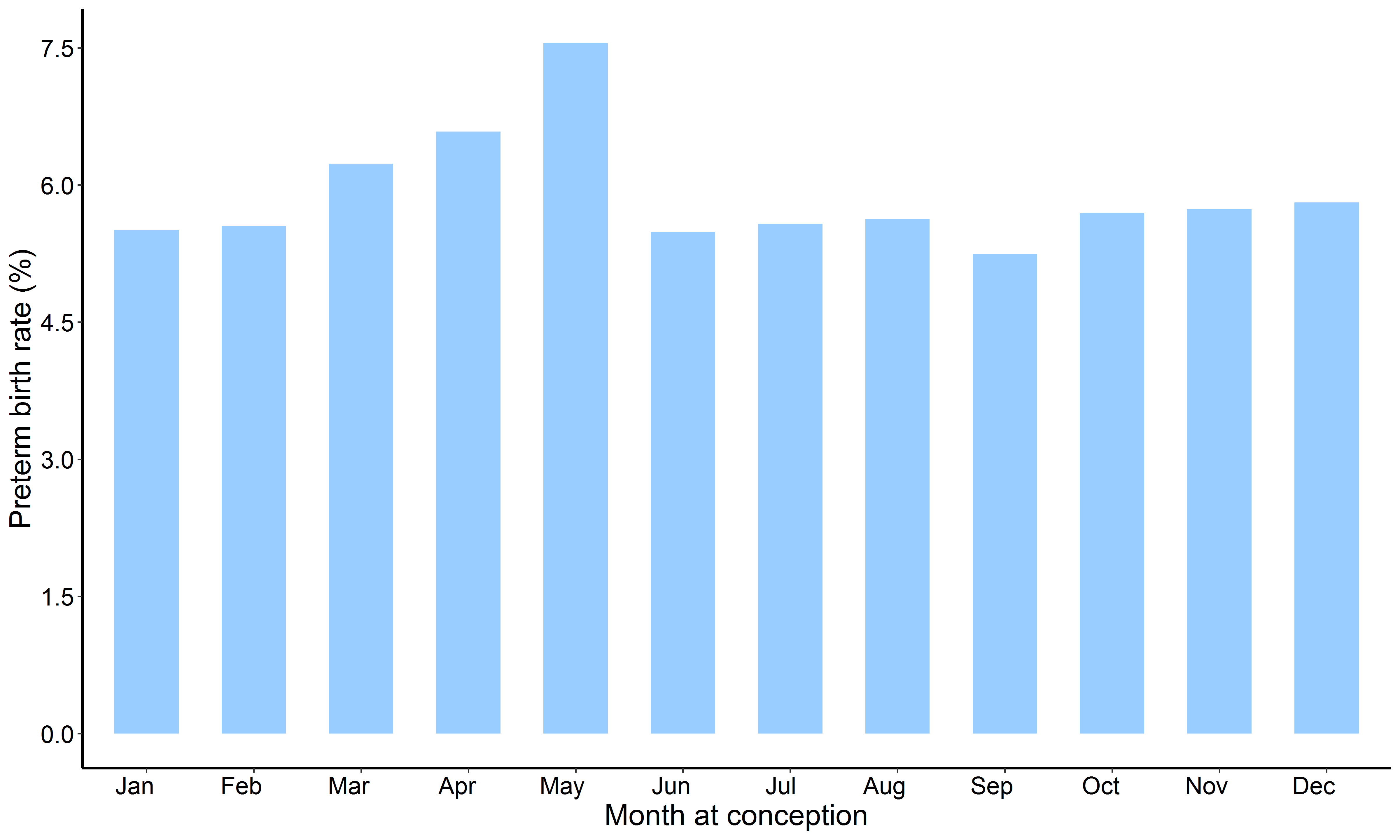


**Figure S2. Rate of preterm birth in each calendar month during 2015-2019** **(before the COVID-19 pandemic)**


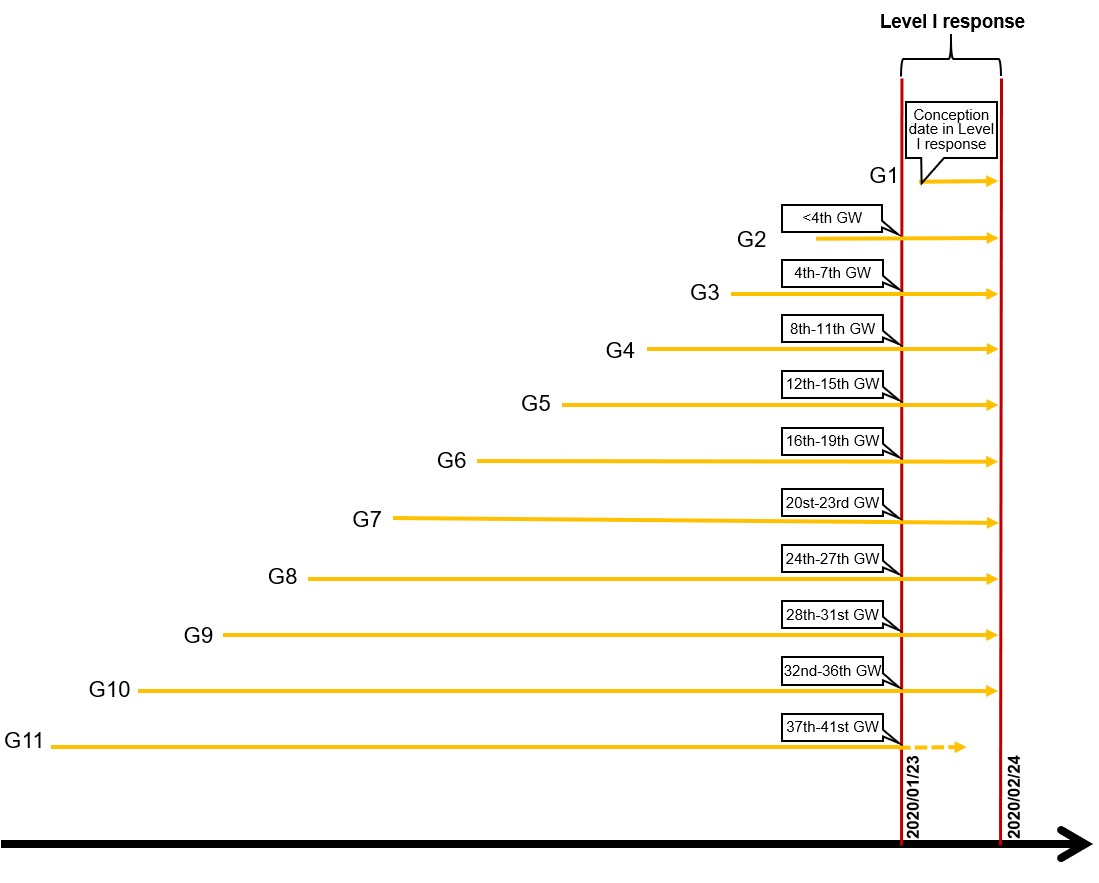


**Figure S3. Division of participants into subgroups according to their GW on January 23^rd^, 2020**

Note: G1-G10: Ten subgroups; 1st-41st GW represent GWs on 1/23/2020.

**
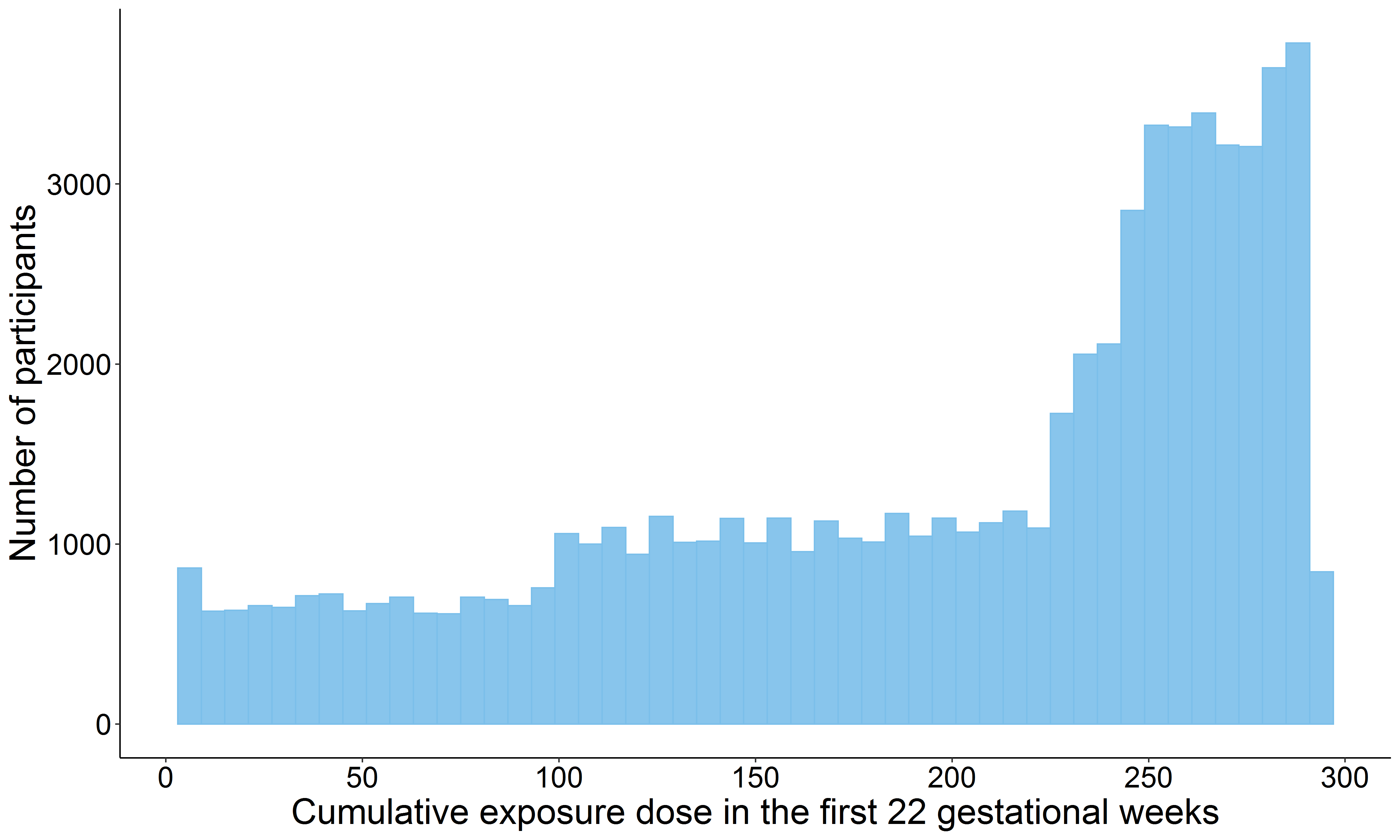
**

**Figure S4. Distribution of the cumulative exposure dose in the first 22 GWs in the exposed group during lockdown**
